# Selection bias may compromise our understanding of the clinical significance of the co-detection of respiratory viruses

**DOI:** 10.1128/spectrum.00397-24

**Published:** 2024-09-19

**Authors:** Justin Z. Amarin, Natasha B. Halasa, Peter F. Rebeiro

**Affiliations:** 1 Division of Pediatric Infectious Diseases, Department of Pediatrics, Vanderbilt University Medical Center, Nashville, Tennessee, USA; 2 Epidemiology Doctoral Program, School of Medicine, Vanderbilt University, Nashville, Tennessee, USA; 3 Division of Infectious Diseases, Department of Medicine, Vanderbilt University Medical Center, Nashville, Tennessee, USA; 4 Division of Epidemiology, Department of Medicine, Vanderbilt University Medical Center, Nashville, Tennessee, USA; 5 Department of Biostatistics, Vanderbilt University Medical Center, Nashville, Tennessee, USA; Barnard College, Columbia University, New York, New York, USA

## LETTER

Stobbelaar et al. compared surrogate measures of disease severity between young children with sole detection of respiratory syncytial virus (RSV) and those with co-detection of RSV and another respiratory virus (1). The authors showed that children with sole RSV detection experienced, on average, more severe illness. They went on to carefully consider multiple explanations, including the clinical significance of detection, temporal correlation of clinical presentation with detection, and immunologic interference. In addition to these explanations, another warrants discussion: selection bias.

In such sentinel surveillance research, particularly for diseases that have mild symptomatology in a sizable proportion of the affected population, the study sample does not represent the general or even source population but rather represents a select subset of children with a more severe disease course requiring medical attention. A relation between study selection and outcome may introduce surveillance bias (a type of selection bias), obscuring valid inferences. If one virus is the sole etiologic culprit, then clinical manifestations must reach some “threshold of virulence” such that medical intervention is warranted. However, in cases of co-detection, this “threshold of virulence” for the selfsame virus is not necessitated. Therefore, comparing children with sole RSV infections to those with RSV co-detection can introduce a false equivalence (2).

For instance, consider a child who contracts a mild case of RSV with symptoms not severe enough to warrant medical attention. If the same child later contracts an adenovirus infection severe enough to provoke symptoms that require medical attention, they become eligible for study inclusion. Comparing this child with another who has been hospitalized for an isolated RSV infection represents a false equivalence. This discrepancy is magnified by the fact that respiratory viruses differ in pathogenic potential. RSV, for example, is typically more virulent in young children than other respiratory viruses (3). Therefore, on average, considering two subgroups of children with RSV co-detection, one in which RSV is the primary driver of illness and another in which it is secondary, illness severity might be “diluted” in the overall group compared with children with sole RSV infection because of the differential virulence of different viruses.

Consider a diametrically opposed scenario in which we compare two groups, one whose members have contracted the least virulent agent currently known and the other with co-detection of that agent alongside some other known agent (which, naturally, would be more virulent). If we compare the two groups in that scenario, it will appear that those with co-detection are, on average, sicker. This regression toward the mean highlights the potential for misleading conclusions if selection bias is not carefully considered. We do not claim that the clinical significance of co-detection can be so neatly summarized; however, selection bias must be carefully considered in the design, analysis, and interpretation of studies of virus–virus co-detection.
